# Engagement With a Web-Based Intervention to Reduce Harmful Drinking: Secondary Analysis of a Randomized Controlled Trial

**DOI:** 10.2196/18826

**Published:** 2020-11-20

**Authors:** Paul U Nordholt, Eva Christalle, Jördis M Zill, Jörg Dirmaier

**Affiliations:** 1 Department of Nursing and Management, Faculty of Business and Social Sciences Hamburg University of Applied Sciences Hamburg Germany; 2 Institute and Outpatient Clinic of Medical Psychology University Medical Center Hamburg-Eppendorf Hamburg Germany

**Keywords:** engagement, usage, alcohol, eHealth, mHealth, readiness to change, self-efficacy, outcome expectancy

## Abstract

**Background:**

Engagement with digital behavior change interventions (DBCIs) is considered a prerequisite for intervention efficacy. However, in many trials on DBCIs, participants use the intervention either only little or not at all.

**Objective:**

To analyze engagement with a web-based intervention to reduce harmful drinking, we explored (1) whether engagement with a web-based alcohol intervention is related to drinking outcomes, (2) which user characteristics are associated with measures of engagement, and (3) whether reported outcomes are associated with data captured by voluntary intervention questionnaires.

**Methods:**

We analyzed data of the intervention arm of a randomized controlled trial on a DBCI to reduce risky alcohol consumption. Data were collected at baseline (T0), after 90 days (T1), and at the end of the 180-day usage period (T2). Engagement with the intervention was measured via system usage data as well as self-reported usage. Drinking behavior was measured as average daily alcohol consumption as well as the number of binge drinking days. User characteristics included demographics, baseline drinking behavior, readiness to change, alcohol-related outcome expectancies, and alcohol abstinence self-efficacy. Following a bivariate approach, we performed two-tailed Welch’s *t* tests and Wilcoxon signed rank/Mann-Whitney *U* tests or calculated correlation coefficients.

**Results:**

The data of 306 users were analyzed. Time spent engaging with the intervention as measured by system usage did not match self-reported usage. Higher self-reported usage was associated with higher reductions in average daily alcohol consumption (T1: ρ=0.39, *P*<.001; T2: ρ=0.29, *P*=.015) and in binge drinking days (T1: ρ=0.62, *P*<.001; T2: ρ=0.3, *P*=.006). Higher usage was reported from users who were single (T1: *P*<.001; T2: *P*<.001), users without children (T1: *P*<.001; T2: *P*<.001), users who did not start or finish secondary education (T1: *P*<.001; T2: *P*<.001), users without academic education (T1: *P*<.001; T2: *P*<.001), and those who worked (T1: *P*=.001; T2: *P*=.004). Relationships between self-reported usage and clinical or psychological baseline characteristics were complex. For system usage, the findings were mixed. Reductions in drinking captured by intervention questionnaires were associated with reported outcomes.

**Conclusions:**

Though self-reported usage could be consistently linked to better outcomes and multiple user characteristics, our findings add to the overall inconclusive evidence that can be found throughout the literature. Our findings indicate potential benefits of self-reports as measures of engagement and intervention questionnaires as a basis for tailoring of intervention content. Future studies should adopt a theory-driven approach to engagement research utilizing psychometrically sound self-report questionnaires and include short ecological momentary assessments within the DBCIs.

**Trial Registration:**

German Clinical Trials Register DRKS00006104; https://tinyurl.com/y22oc5jo

## Introduction

In many trials on self-guided digital behavior change interventions (DBCIs), a substantial proportion of participants use the intervention only little or not at all [1]. This phenomenon might limit the validity of trial results, since some form of engagement with the intervention is assumed to be a condition for its efficacy [2]. Therefore, it is considered good practice to describe how engagement was measured and report usage along with the primary outcomes when conducting a randomized controlled trial (RCT) on a DBCI [3].

Most likely due to the interdisciplinarity of the field, despite efforts to find a common language [4], the terms engagement, adherence, dose, and usage are often used interchangeably in the literature, and different theoretical perspectives on the topic have emerged [5]. Perski et al [6] found that the behavioral sciences focus on quantitative aspects of engagement, conceptualizing it as the amount of usage over time. However, in computer science literature, research on engagement with DBCIs focuses on its qualitative aspects. In defining engagement as “[…] (1) the extent (eg, amount, frequency, duration, depth) of usage and (2) a subjective experience characterized by attention, interest, and affect,” Perski et al [6] highlight the importance of both aspects to obtain a better understanding of engagement and its relation to behavior change. Furthermore, Yardley et al [1] argue that mere engagement with the intervention content is not sufficient to bring about behavior change. Rather, after a phase of engagement with the intervention content, individuals must actively engage in behavior change to achieve the desired outcomes.

In the majority of studies on eHealth and mobile health interventions, engagement is operationalized as the actual usage of the intervention and is measured based on system usage data (SUD) [7]. SUD includes but is not limited to the number of logins, number of modules completed, and the amount of time spent in the intervention. While these measures seem to be predictive of outcomes for interventions targeting physical health, findings for mental health interventions are mixed [8]. Concerning interventions aiming to reduce harmful alcohol consumption, there is some evidence for engagement-outcome relationships [9,10]. However, some studies could not find an association between engagement and the extent of alcohol reduction [11]. It has been argued that the overall inconclusive findings might be due to a lack of consensus on how to measure engagement [8] or different patterns of usage [7]. The latter claim is supported by Milward et al [12] who found that some users of a smartphone app aimed at reducing harmful drinking used it to track their alcohol consumption, while others also used its other features. Furthermore, individuals might differ with respect to the extent of intervention usage required for them to engage in behavior change [1]. Discontinuing the use of an intervention might therefore not be necessarily linked to worse outcomes. It can also mean that the individual’s information needs are satisfied and they are already engaged in changing their behavior. A person showing high intervention usage, on the other hand, might have trouble transferring intervention content to actual behavior change and may keep on looking for support in the intervention content. SUD can only capture aspects of the micro level of engagement with the intervention; however, the macro level of engagement with behavior change is a necessity for improved outcomes. Therefore, Yardley et al [1] recommend the utilization of multiple measures of engagement. A cost-efficient way to capture different aspects of engagement are self-report questionnaires. However, at this point, only few of such questionnaires are available and have been psychometrically evaluated [7,13].

As noted above, some form of engagement is necessary to achieve behavior change. To further increase the reach and impact of DBCIs, it is important to identify factors that influence engagement. To date, various user characteristics have been proposed to be related to engagement with intervention content, and overall, the evidence is puzzling [14]. A systematic review identified female gender and a higher treatment expectancy as predictive of higher engagement. Findings for age and baseline problem severity were mixed [15]. Concerning DBCIs targeting alcohol consumption, higher age, higher education, and female gender were found to predict higher engagement [11,16,17]. For baseline alcohol consumption, positive and negative associations with engagement have been found, while some studies could not establish such a relationship [11,17]. Concerning psychological variables, higher motivation for treatment and behavior change as well as lower confidence in one’s own ability to change were found to be associated with higher engagement [16,17].

To improve engagement and consequently intervention outcomes, different approaches have been suggested. One of them is tailoring. Tailoring seems to be a desired and efficient way to increase engagement for different types of interventions [15,18,19]. Dynamic tailoring of intervention content relies on the user providing information at some point of the interaction with the DBCI. Based on this user input, the personalized content can be delivered automatically by the system. Apart from static variables such as age or gender, it seems reasonable to consider changes in the target behavior as a basis for tailoring of content. Based on such data, those users who have already changed their behavior could receive content concerning relapse prevention. For others who are still struggling, additional content on behavior change techniques or motivational content could be provided. However, to our knowledge, it is yet unclear whether data captured by intervention questionnaires corresponds to reported outcomes.

Based on data collected between 2015 and 2017 in an RCT on a web-based intervention to reduce risky alcohol consumption—Vorvida [20]—we analyzed engagement with the intervention. To do so, we explored the following 3 questions.

*Engagement and outcomes*: How is engagement with the intervention related to drinking outcomes?

*User characteristics and engagement*: Which user demographics and clinical or psychological baseline characteristics are associated with measures of engagement?

*Intervention questionnaires and outcomes*: Are reported outcomes associated with data captured by voluntary intervention questionnaires?

As much as possible, post hoc analyses were guided by the AMUsED (Analyzing and Measuring Usage and Engagement Data) Framework [21].

## Methods

### RCT Details

For detailed information on the study design and procedure of the RCT as well as the intervention, see the publication of the results [20] and the study protocol for the RCT [22]. The RCT was registered at the German Clinical Trials Register (reference number: DRKS00006104), and ethical approval was obtained from the Ethics Committee of the Hamburg State Chamber of Physicians (reference number: PV4802).

### Study Design and Procedure

The data we explored in this study were collected from the intervention group of a parallel group pragmatic RCT at 3 time points: at baseline (T0) as well as at 3 months (T1) and 6 months (T2) later. Because access to the intervention was granted for a period of 180 days, T1 corresponds to the middle and T2 corresponds to the end of the usage period. Participants were recruited via health care providers (eg, outpatient centers, information centers, family doctor’s offices) in northern Germany and German online and offline media (internet self-help forums, newspaper advertisements). Inclusion criteria were informed consent, a minimum age of 18 years, and either an average consumption of at least 12/24 g (women/men) of pure alcohol per day or an AUDIT-C (Alcohol Use Disorders Identification Test-Concise) score of at least 3 or both.

After filling out a screening questionnaire at the study website, within 1 week, participants received an email informing them whether inclusion criteria were met. Included participants received a link to the baseline questionnaire and were asked to respond. Right after baseline assessment, participants were randomly allocated to either the intervention or the care as usual/waitlist group. A centralized, software-driven, computerized, simple randomization procedure was used so that randomization could not be subverted by the team of researchers and concealed allocation was ensured. Participants in the intervention group received an access key and a link to the intervention. After registration, the program was activated for 180 days. To encourage usage, participants in the intervention group received an email reminder at 3, 6, 9, and 16 weeks after receiving the access key. Six months after the T2 assessment, participants in the care as usual/waitlist group gained access to the intervention.

### Intervention

Vorvida is an unguided web-based intervention, which was designed for persons older than 18 years who consider their alcohol consumption patterns to be problematic. The intervention content is tailored to the user depending upon his/her answers in simulated dialogues. Vorvida is based primarily on principles and techniques used in cognitive behavioral therapy. It incorporates different techniques to change behavior and cognition, eg, motivational interviewing, cognitive restructuring, or mindfulness-based methods.

Vorvida is organized in 4 modules. The first module is concerned with individual drinking patterns, motivation to change, and goal setting. The second module focuses on cognitive and mindfulness-based methods to cope with alcohol cravings. The third module informs about coping with risk situations, while the fourth module content is based on relapse prevention. Three short questionnaires are embedded within the program, and users are encouraged to respond to these regularly (self-monitoring). Vorvida can be accessed via standard internet browsers on any desktop, laptop, or mobile computers, including smartphones. Font and image sizes adjust automatically to different screen sizes (responsive design). In addition, Vorvida offers homework materials in the form of PDFs to print and use outside of the intervention.

### Measures

#### Details

In the following section, we briefly describe the measures used, including the abbreviations we will use to refer to them. An overview of all measures used in the analyses, including a short description as well as information on when the assessment took place and references to additional information [23-27], are given in Multimedia Appendix 1.

#### Drinking Behavior

Drinking behavior was measured in 2 ways: first, as the average daily alcohol consumption in grams of alcohol within the last 7 days by the Timeline Followback (TFB) method [23]; second, as the number of binge drinking days (BDDs) within the last 30 days by asking on how many days the participants drank 5 or more alcoholic beverages on 1 occasion. For research questions (1) and (3), we calculated the individual change scores.

#### Engagement

Engagement was operationalized as usage of the intervention and measured by SUD and self-report. SUD included the frequency of the utilization of the intervention questionnaires and the time spent in the intervention measured as five-minute blocks of activity (FMB). The FMB was calculated based on server logs. To avoid overestimation of time spent in the intervention, if a user did not create an event (eg, opening a new page) within a time-frame of 5 minutes, he/she was considered inactive. Three different questionnaires were included in the intervention: a daily alcohol check (DAC), assessing the number of standard units of alcoholic drinks consumed on the day before, a weekly alcohol check (WAC) based on the AUDIT-C [28], and “a mood check” (MC) (affective checklist). As an indicator for the breadth of usage, we included the frequency of use of these questionnaires in our analyses. All SUD were measured continuously and were provided by the GAIA AG—the company responsible for the development and deployment of Vorvida. Self-reported usage (SRU) was assessed at T1 and T2 by asking the participants to rate on a 6-point scale how often they used Vorvida in the past 3 months.

#### Sociodemographic Data

Sociodemographic data were collected at T0 and these data included the participants’ age, gender (female/male), relationship status (single/nonsingle), children (no children/one or more children), education (general higher education entrance qualification [Abitur]/no general higher education entrance qualification [no Abitur]), job qualification (academic studies/no academic studies), and job status (working/not working).

#### Baseline User Characteristics

Baseline user characteristics consisted of psychological constructs as well as clinical measures concerning drinking behavior. Clinical measures were the age of the first alcohol consumption, the age of regular alcohol consumption, average daily alcohol consumption in grams of alcohol, number of BDDs, and the number of drinking days [23] at baseline (T0). Additionally, we assessed the motivation to change with a readiness ruler [24] and readiness to change with the Readiness to Change Questionnaire [27]. Alcohol abstinence self-efficacy was measured using the Alcohol Abstinence Self-Efficacy Scale [25] and alcohol expectancies were assessed with the Comprehensive Alcohol Expectancy Questionnaire [26].

### Data Preparation

Since we were interested in the relationship between usage and changes in drinking behavior, we calculated individual change scores for reductions in the TFB and BDD between T0 and T1 (T1) and between T1 and T2 (T2) as well as total reductions (TOT). We calculated the frequency of utilization of the intervention questionnaires and the time spent in the intervention for the same intervals. To determine whether changes in the DAC or WAC predicted changes in the drinking behavior at T1 or T2, we calculated values for these questionnaires as follows: (1) baseline value: DAC, mean of the individual scores within 14 days starting with the first utilization, and WAC, individual score of the first utilization; and (2) 30-day value/90-day value: DAC, mean of the individual scores within 14 days starting at 30/90 days after the first utilization, and WAC, individual score closest to 30/90 days after the first utilization within a range of +/-10 days

### Inferential Statistics

Since, as of yet, research findings concerning our research questions are inconclusive, modeling relationships among the several variables in complex ways appeared inadvisable. Therefore, we chose a bivariate approach. When the independent variable was binary, we performed two-tailed Welch’s *t* tests when the dependent variable was metrically scaled and Wilcoxon signed rank/Mann-Whitney *U* tests when it was ordinally scaled. When both the independent variable and the dependent variable were at least ordinally scaled, we calculated Pearson correlation coefficients or Spearman correlation coefficients, respectively. For each research question, tests were performed on an alpha level of 5%. Holm-corrected *P* values are reported.

## Results

### Summary

After a brief description of the baseline characteristics of our sample and the general usage behavior, the results of the analyses concerning our research questions are shown below.

### Baseline Characteristics

The statistical analyses were conducted on a final sample of the 306 participants (females, n=170, 55.6%) of the intervention group, who completed the assessments at all 3 time points. The age range of these participants was 18 to 69 years with a mean (SD) age of 40.36 (11.15) years. Of the 306 participants, 128 (41.8%) reported to be in a relationship and 92 (30.1%) reported having at least one child. A total of 119 (38.9%) participants reported achieving a general higher education entrance qualification (Abitur), and 78 (25.5%) finished academic studies. A total of 259 (86.6%) participants were working at the time of the baseline assessment. Further information on the baseline demographics and user characteristics are shown in Table 1.

**Table 1:** Overview of the baseline characteristics of the final sample.

Measure	Participants (n)	Mean (SD)	Median (range)
Age (years)	306	40.36 (11.15)	40 (18-69)
AFC^a^ (years)	305	15.04 (3.23)	15 (2-45)
ARC^b^ (years)	305	17.5 (4.21)	17 (0-45)
QFI-days^c^	306	23.05 (8.31)	27 (0-30)
TFB^d^	306	52.91 (56.68)	41.64 (0-515.71)
BDD^e^	304	16.79 (11.45)	20 (0-30)
RR-I^f^	296	8.11 (1.77)	8 (1-10)
RR-C^g^	296	6.08 (2.44)	6 (1-10)
AASE-T^h^	298	62.26 (15.22)	60 (23-93)
AASE-C^i^	296	55.82 (16.1)	59 (20-98)
CAEQ-A^j^	299	7.69 (3.18)	8 (3-15)
CAEQ-CP^k^	299	11.64 (3.1)	12 (4-20)
CAEQ-SE^l^	299	8.19 (3.02)	9 (3-15)
CAEQ-SP^m^	299	16.28 (3.96)	16 (5-25)
CAEQ-T^n^	299	13.21 (2.89)	13 (4-20)
RCQ-C^o^	298	14.54 (3.57)	14 (4-20)
RCQ-P^p^	298	8.7 (3.47)	9 (4-19)
RCQ-A^q^	298	12.2 (3.43)	12 (4-20)

^a^AFC: age of first alcohol consumption.

^b^ARC: age of regular alcohol consumption.

^c^QFI-Days: drinking days measured with the Quantity-Frequency Index.

^d^TFB: average daily alcohol consumption measured with the Timeline Followback approach.

^e^BDD: binge drinking day.

^f^RR-I: readiness ruler importance scale.

^g^RR-C: readiness ruler confidence scale.

^h^AASE-T: Temptation scale of the Alcohol Abstinence Self-Efficacy Scale.

^i^AASE-C: Confidence scale of the Alcohol Abstinence Self-Efficacy Scale.

^j^CAEQ-A: Aggression scale of the Comprehensive Alcohol Expectancy Questionnaire.

^k^CAEQ-CP: Cognitive impairment and physical discomfort scale of the Comprehensive Alcohol Expectancy Questionnaire.

^l^CAEQ-SE: Sexual enhancement scale of the Comprehensive Alcohol Expectancy Questionnaire.

^m^CAEQ-SP: Social assertiveness and positive affect scale of the Comprehensive Alcohol Expectancy Questionnaire.

^n^CAEQ-T: Tension reduction scale of the Comprehensive Alcohol Expectancy Questionnaire.

^o^RCQ-C: Contemplation scale of the Readiness to Change Questionnaire.

^p^RCQ-P: Precontemplation scale of the Readiness to Change Questionnaire.

^q^RCQ-A: Action scale of the Readiness to Change Questionnaire.

### Measures of Engagement

While 61 of the total 306 (19.9%) participants did not log in to the website even once, only 6 of the 183 (3.3%) participants who reported their usage at T2 reported not having used the intervention at all. At T1, the most chosen answer (73/200, 36.5%) for the SRU was “weekly 3-4 hours,” which would approximately be at least 2160 minutes in a 90-day period. However, based on the FMB, the maximum time spent in the intervention within the first 90 days was 845 minutes. Table 2 shows that, after this period, in the following 90 days (T2) of the 180-day usage period, the usage of the intervention declined for all system usage metrics. Concerning the intervention questionnaires, we found that the MC questionnaire was used less often than the DAC as well as the weekly AUDIT-C. For an overview of the usage statistics, see Table 2.

**Table 2:** Overview of the engagement with the digital behavior change intervention as captured by all measures available.

Measures and metrics		T1^a^	T2^b^	TOT^c^
**Five-minute blocks of activity**				
	Minimum	0	0	0
	Median (1st quartile, 3rd quartile)	205 (30, 275)	20 (0, 25)	240 (30, 295)
	Mean (SD)	184.4 (150.3)	25.15 (55.71)	209.5 (184.64)
	Maximum	845	460	1105
	Participants (N)	306	306	306
**Daily alcohol check frequency**				
	Minimum	0	0	0
	Median (1st quartile, 3rd quartile)	22 (1, 23)	3 (0, 3)	25 (1, 26)
	Mean (SD)	16.77 (15.89)	3.72 (10.04)	20.49 (23.29)
	Maximum	83	90	155
	Participants (N)	306	306	306
**Weekly alcohol check frequency**				
	Minimum	0	0	0
	Median (1st quartile, 3rd quartile)	12 (1, 13)	1 (0, 1)	13 (1, 14)
	Mean (SD)	7.94 (5.81)	1.11 (2.31)	9.05 (7.05)
	Maximum	16	25	37
	Participants (N)	306	306	306
**Mood check frequency**				
	Minimum	0	0	0
	Median (1st quartile, 3rd quartile)	1 (1, 4)	0 (0, 0)	1 (1, 4)
	Mean (SD)	5.98 (12.9)	1.12 (5.47)	7.1 (17.05)
	Maximum	80	68	147
	Participants (N)	306	306	306
**Self-reported usage**				
	Minimum	0	0	N/A^d^
	Median (1st quartile, 3rd quartile)	3 (2, 4)	3 (2, 3)	N/A
	Mean (SD)	2.82 (1.19)	2.59 (1.12)	N/A
	Maximum	5	4	N/A
	Participants (N)	200	183	N/A

^a^T1: period from baseline to first post-assessment (90 days).

^b^T2: period from first to second post-assessment (90 days).

^c^TOT: total usage period (180 days).

^d^N/A: not applicable.

### Engagement and Outcomes

All SUD except the MC count at T2 and the WAC count at T1 were correlated with one another, with coefficients between r=0.26 and r=0.98. SRU T1 was significantly positively correlated with FMB T1 (ρ=0.29, *P*=.005), FMB TOT (ρ=0.3, *P*=.003), DAC T1 (ρ=0.29, *P*=.007), and WAC T1 (ρ=0.44, *P*<.001). WAC T1 was also positively correlated with SRU T2 (ρ=0.38, *P*<.001). However, the MC was negatively correlated with SRU T1 and SRU T2 for all 3 intervals with coefficients between ρ=–0.26 and ρ=–0.34.

Significant correlations between changes in drinking behavior and SUD were rare. Participants who utilized the WAC more frequently within the first 90 days (T1) appeared to have achieved higher reductions in BDDs within this period (r=0.26, *P*=.03) as well as in total (r=0.36, *P*<.001). However, more frequent use of the WAC after the first 90 days was associated with smaller total reductions in BDDs (r=–0.28, *P*=.02). A negative relationship between the total reductions in BDDs was also found for MC T1 (r=–0.39, *P*<.001) and MC TOT (r=–0.37, *P*<.001). Changes in average daily alcohol consumption (TFB) were not significantly correlated with SUD.

In contrast to the SUD, both SRU T1 and SRU T2 were associated with changes in TFB as well as in BDDs. SRU at T1 was positively correlated with BDDs T1 (ρ=0.62, *P*<.001), BDD TOT (ρ=0.65, *P*<.001), TFB T1 (ρ=0.39, *P*<.001), and TFB TOT (ρ=0.42, *P*<.001). SRU T2 was associated with BDD T1 (ρ=0.27, *P*=.03), BDD T2 (ρ=0.3, *P*=.006), BDD TOT (ρ=0.42, *P*<.001), and TFB T2 (ρ=0.29, *P*=.015). This indicates that those participants who reported higher usage also reported higher reductions in BDDs as well as in average daily alcohol consumption. A table including all bivariate correlations can be found in Multimedia Appendix 2.

### Baseline User Characteristics and Engagement

We first described the results of the analyses concerning relationships between sociodemographic baseline characteristics and follow-up with the results of the analyses on clinical and psychological baseline characteristics.

#### Sociodemographic User Characteristics

User age was not correlated with usage. Neither Pearson correlation coefficients for age and FMB at T1 (r=0.16, *P*=.09) and FMB at T2 (r=0.11, *P*=.82) nor the Spearman coefficients for age and SRU at T1 (ρ=–0.13, *P*=.82) and SRU T2 (ρ=–0.2; *P*=.11) were significantly different from zero after Holm-correction of *P* values.

The only significant differences on the system usage measure (FMB) were found between singles and nonsingles (*P*=.02) at T1 as well as between those who did not complete secondary education and those who did (*P*=.02) at T1 (Table 3).

However, for the subjective measure, for every demographic, except gender, the groups differed significantly (Table 3). Higher usage was reported from singles (vs nonsingles) (T1: W=6883.5, *P*<.001; T2: W=4832, *P*<.001), users without children (vs with one child or more) (T1: W=5003, *P*<.001; T2: W=3801.5, *P*<.001), users who did not start or finish secondary education (vs with secondary education) (T1: W=6596.5, *P*<.001; T2: W=5300, *P*<.001), users without academic education (vs academic education) (T1: W=4622.5, *P*<.001, W=3656, *P*<.001) and those who worked (vs no work) (T1: W=1272.5, *P*=.001; T2: W=774, *P*=.004) at T1 and T2. These results must be interpreted cautiously because group sizes tended to differ substantially.

**Table 3:** Relationships between sociodemographic user characteristics and measures of engagement.

Measures, Groups		Mean (SD)	*t* (df)^a^	W^b^	*P* value^c^
**FMB^d^ T1^e^, Gender**			0.44 (295.52)	N/A^f^	>.99
	Females (n=170)	187.74 (154.11)			
	Males (n=136)	180.18 (145.86)			
**FMB T2^g^, Gender**			–0.13 (291.19)	N/A	>.99
	Females (n=170)	24.76 (56.17)			
	Males (n=136)	25.63 (55.35)			
**SRU^h^ T1, Gender**			N/A	5075.5	>.99
	Females (n=113)	2.78 (1.24)			
	Males (n=87)	2.87 (1.12)			
**SRU T2, Gender**			N/A	4563	>.99
	Females (n=102)	2.5 (1.12)			
	Males (n=81)	2.7 (1.1)			
**FMB T1, Relationship status**			3.31 (198.94)	N/A	.02
	Single (n=178)	209.80 (115.76)			
	Nonsingle (n=128)	149.02 (182.79)			
**FMB T2, Relationship status**			–0.37 (154.48)	N/A	>.99
	Single (n=178)	24.02 (30.36)			
	Nonsingle (n=128)	26.72 (78.52)			
**SRU T1, Relationship status**			N/A	6883.5	<.001
	Single (n=142)	3.24 (0.99)			
	Nonsingle (n=58)	1.79 (1.00)			
**SRU T2, Relationship status**			N/A	4832	<.001
	Single (n=140)	2.89 (.93)			
	Nonsingle (n=43)	1.61 (1.09)			
**FMB T1, With/Without children**			1.39 (128.68)	N/A	>.99
	No children (n=214)	193.41 (129.18)			
	One or more children (n=92)	163.37 (189.78)			
**FMB T2, With/Without children**			–0.07 (127.66)	N/A	>.99
	No children (n=214)	24.98 (47.78)			
	One or more children (n=92)	25.54 (71.14)			
**SRU T1, With/Without children**			N/A	5003	<.001
	No children (n=159)	3.05 (1.12)			
	One or more children (n=41)	1.93 (1.01)			
**SRU T2, With/Without children**			N/A	3801.5	<.001
	No children (n=151)	2.8 (1.03)			
	One or more children (n=32)	1.63 (0.98)			
**FMB T1, Educational qualification**			–3.38 (200.88)	N/A	.02
	No Abitur (n=187)	208.57 (129.06)			
	Abitur (n=119)	146.39 (172.49)			
**FMB T2, Educational qualification**			–0.004 (176.6)	N/A	>.99
	No Abitur (n=187)	25.16 (43.94)			
	Abitur (n=119)	25.13 (70.57)			
**SRU T1, Educational qualification**			N/A	6596.5	<.001
	No Abitur (n=145)	3.21 (.99)			
	Abitur (n=55)	1.78 (1.03)			
**SRU T2, Educational qualification**			N/A	5300	<.001
	No Abitur (n=138)	2.95 (.92)			
	Abitur (n=45)	1.49 (.92)			
**FMB T1, Job qualification**			–2.93 (115.15)	N/A	.07
	Nonacademic field (n=228)	200.26 (140.12)			
	Academic field (n=78)	137.95 (169.33)			
**FMB T2, Job qualification**			0.48 (93.81)	N/A	>.99
	Nonacademic field (n=228)	23.99 (44.58)			
	Academic field (n=78)	28.53 (80.13)			
**SRU T1, Job qualification**			N/A	4622.5	<.001
	Nonacademic field (n=166)	3.06 (1.08)			
	Academic field (n=34)	1.65 (.98)			
**SRU T2, Job qualification**			N/A	3656	<.001
	Nonacademic field (n=153)	2.79 (1.02)			
	Academic field (n=30)	1.57 (1.01)			
**FMB T1, Job status**			–0.87 (55.48)	N/A	>.99
	No work (n=47)	206.06 (191.52)			
	Work (n=259)	180.44 (141.65)			
**FMB T2, Job status**			–1.09 (52.93)	N/A	>.99
	No work (n=47)	36.17 (79.13)			
	Work (n=259)	23.15 (50.27)			
**SRU T1, Job status**			N/A	1272.5	.001
	No work (n=28)	2 (.94)			
	Work (n=172)	2.95 (1.17)			
**SRU T2, Job status**			N/A	774	.004
	No work (n=19)	1.68 (1)			
	Work (n=164)	2.7 (1.08)			

^a^Welch’s *t* test.

^b^Wilcoxon signed rank/Mann-Whitney *U* tests.

^c^*P* values are Holm-corrected.

^d^FMB: five-minute blocks of activity.

^e^T1: period from baseline to first post-assessment (90 days).

^f^N/A: not applicable.

^g^T2: period from first to second post-assessment (90 days).

^h^SRU: self-reported usage.

#### Clinical and Psychological User Characteristics

Concerning SUD, only more drinking days (r=0.29, *P*<.001) and more BDDs (r=0.27, *P*<.001) were associated with more time spent using the intervention within the first 90 days (T1). However, for the self-report measure, multiple correlation coefficients were significantly different from zero. Users with a higher number of drinking days reported higher usage within the first (T1: ρ=0.37, *P*<.001) and second (T2: ρ=0.6, *P*<.001) 90 days of access to the intervention. The same was true with regard to the number of BDDs (T1: ρ=0.52, *P*<.001; T2:ρ=0.56, *P*<.001). Those with an older age when they first consumed alcohol reported higher usage at both T1 (ρ=0.34, *P*<.001) and T2 (ρ=0.43, *P*<.001).

Higher scores on the temptation scale of the Alcohol Abstinence Self-Efficacy Scale were significantly positively correlated with higher reported usage at T2 (ρ=0.46, *P*<.001) but not at T1 (ρ=0.19, *P*>.99). From the comprehensive alcohol expectancy questionnaire, the aggression scale was significantly correlated with SRU at T1 (ρ=0.34, *P*<.001) and T2 (ρ=0.47, *P*<.001). The same was true for the sexual enhancement scale of the comprehensive alcohol expectancy questionnaire (T1: ρ=0.38, *P*<.001; T2: ρ=0.54, *P*<.001). Higher scores on the confidence scale of the readiness ruler were associated with higher reported usage at T1 (ρ=0.26, *P*=.049). While lower scores on the contemplation (T1: ρ=–0.43, *P*<.001; T2: ρ=–0.31, *P*=.008) and higher scores on the precontemplation scale (T1: ρ=0.57, *P*<.001; T2: ρ=0.43, *P*<.001) of the readiness to change questionnaire were associated with higher SRU at T1 and T2, no significant association was found for scores on the action scale (T1: ρ=–0.07, *P*>.99; T2: ρ=0.09, *P*>.99). All correlations that were calculated can be found in Multimedia Appendix 3.

### Intervention Questionnaires and Outcomes

For the 30-day period, only changes in the WAC scores were correlated with total reductions in the TFB (r=0.24, *P*=.015). However, for the 90-day period, changes in WAC scores were correlated with changes in TFB at T1 (r=0.31, *P*<.001) as well as with total changes (r=0.39, *P*<.001) and changes in the DAC were positively correlated with all 4 outcome measures with coefficients between r=0.34 and r=0.58 (Table 4).

**Table 4:** Correlations between intervention questionnaires and assessment questionnaires.

Measures		WAC^a^ 30D^b^	WAC 90D	DAC^c^ 30D	DAC 90D	TFB^d^ T1^e^	TFB TOT^f^	BDD^g^ T1	BDD TOT
**WAC 30D**									
	n^h^	200	168	189	170	200	200	175	169
	*r*	1	0.69	0.31	0.16	0.14	0.24	0.01	–0.04
	*P* value	<.001	<.001	<.001	.66	.73	.02	>.99	>.99
**WAC 90D**									
	n	168	169	165	165	165	169	163	159
	*r*	0.69	1	0.35	0.34	0.31	0.39	0.23	0.18
	*P* value	<.001	<.001	<.001	<.001	.001	<.001	.06	.48
**DAC 30D**									
	n	189	165	191	169	191	191	170	164
	*r*	0.31	0.35	1	0.24	0.04	–0.02	0.08	0.01
	*P* value	<.001	<.001	<.001	.03	>.99	>.99	>.99	>.99
**DAC 90D**									
	n	170	165	169	173	173	173	165	161
	*r*	0.16	0.34	0.24^i^	1	0.34	0.36	0.58	0.51
	*P* value	.66	<.001	.03	<.001	<.001	<.001	<.001	<.001
**TFB T1**									
	n	200	165	191	173	306	306	204	189
	*r*	0.14	0.31	0.04	0.34	1	0.84	0.42	0.37
	*P* value	.73	.001	>.99	<.001	<.001	<.001	<.001	<.001
**TFB TOT**									
	n	200	169	191	173	306	306	204	187
	*r*	0.24	0.39	–0.02	0.36	0.84	1	0.33	0.58
	*P* value	.02	<.001	>.99	<.001	<.001	<.001	<.001	<.001
**BDD T1**									
	n	175	163	170	165	204	204	204	189
	*r*	0.01	0.23	0.08	0.58	0.42	0.33	1	0.68
	*P* value	>.99	.06	>.99	<.001	<.001	<.001	<.001	<.001
**BDD TOT**									
	n	169	159	164	161	189	187	189	189
	*r*	–0.04	0.18	0.01	0.51	0.37	0.58	0.68	1
	*P* value	>.99	.48	>.99	<.001	<.001	<.001	<.001	<.001

^a^WAC: weekly alcohol check.

^b^D: time span from baseline in days.

^c^DAC: daily alcohol check.

^d^TFB: average daily alcohol consumption measured with the Timeline Followback approach.

^e^T1: period from baseline to first post-assessment (90 days).

^f^TOT: total usage period (180 days).

^g^BDD: binge drinking day.

^h^Associated number of cases.

^i^The correlation was significant at a significance level of .05 (two-tailed).

## Discussion

### Principal Findings

To analyze engagement with Vorvida, we explored the following 3 questions.

*Engagement and outcomes*: How is engagement with the intervention related to drinking outcomes?

*User characteristics and engagement*: Which user demographics and clinical or psychological baseline characteristics are associated with measures of engagement?

*Intervention questionnaires and outcomes*: Are reported outcomes associated with data captured by voluntary intervention questionnaires?

We used SUD as well as SRU to measure engagement. The amount of time spent engaging with the intervention as measured by SUD did not match the SRU. Users tended to overestimate their actual usage of the intervention. This is in line with the general observation that self-reports of internet and smartphone use tend to be inaccurate [29,30]. More specifically, Perski et al [31] showed that the amount of usage of a smartphone app for reducing alcohol consumption as measured by self-report did not correlate with server log data. However, in our study, at least for the first 90 days of intervention usage, both measures correlated positively with one another. After the first 90 days, average engagement with the intervention declined on all measures used.

### Engagement and Outcomes

Similar to that reported in other studies [8,10,11], no conclusive relationships could be established between engagement as measured by SUD and changes in drinking behavior. While the time spent using the intervention as measured by SUD was not associated with changes in drinking behavior, the results concerning relationships between the frequency of the utilization of the intervention questionnaires and changes in drinking behavior remained inconclusive. However, SRU was correlated positively with reductions in average daily alcohol consumption as well as reductions in BDDs.

An explanation for this phenomenon, following Yardley et al [1], would be that self-reports capture aspects of engagement with behavior change, apart from the actual usage of the website. When asked to rate how often they used Vorvida, apart from the actual time spent on the website, time spent engaging with printed homework material and in applying newly learned techniques to real-life situations might have influenced the participants’ choices. It seems reasonable to assume that those participants who spent more time and effort actually implementing the newly learned skills would report higher drinking reductions as well as higher usage. Since the SUD does not capture time spent engaging with behavior change, this might also explain why SRU was overall higher than usage captured by the SUD.

### User Characteristics

Regarding user demographics, except age and gender, all other measures were associated with SRU. Higher usage was reported from singles, users without children, users without secondary education, as well as users without academic education, and users who work. With regard to the time spent using the intervention as captured by server logs, significantly higher usage was recorded only for singles and users without secondary education.

The facts that in our analyses, neither age nor gender were associated with higher engagement and participants with higher education engaged less with the intervention than those with lower education levels stand in contrast to previous findings [11,16,17]. In addition to this, we found that singles and users without children tended to engage more with the intervention, though only tests for differences in the self-report measures were significant. Nonetheless, one could argue that these associations are due to singles and persons without children having more free time to use the intervention and higher educated users needing less time to understand and implement the intervention content.

Concerning clinical and psychological baseline characteristics, we also found multiple associations with SRU, while only the number of drinking days and BDDs were positively correlated with higher SUD during the first usage period. In addition to more drinking days and more BDDs, first consumption at an older age was associated with higher SRU. Since we found more drinking days and more BDDs at baseline to be positively related to engagement but no relationship for average daily alcohol consumption, our results on baseline problem severity add to the mixed findings of previous studies [11,17].

Participants reporting more temptation to drink alcohol in different situations also reported higher usage at T2 but not at T1. However, confidence to withstand drinking in these situations was not associated with reports of usage. Unlike the findings of Murray et al [16], we found higher confidence in one’s ability to change to be associated with higher engagement. Participants who were more confident in their ability to achieve a reduction reported higher engagement within the first usage period. With respect to alcohol expectations, only those related to aggression or sexual enhancement were correlated with engagement. Interestingly, higher scores on the precontemplation scale and lower scores on the contemplation scale were associated with higher SRU. This suggests that those participants who were already thinking about reducing their drinking, used the intervention less than those who had no intention to change. Since individuals in the precontemplation stage are not intending to change their drinking yet [29], one would expect them to engage less with the intervention than those already contemplating about changing. Consistent with this assumption, previous studies found readiness to change to be associated with higher engagement [17]. However, our results point in a different direction. While higher precontemplation scores were associated with higher engagement, the opposite was true for contemplation scores.

Since all clinical and psychological measures related to engagement (except confidence to achieve behavior change) were correlated with one another, an underlying factor might be responsible for their relationship with SRU. A potential factor that comes to mind is impulsivity. Personality traits related to impulsivity have been robustly linked to alcohol use, especially drinking frequency, binge drinking, and alcohol-related problem behavior [32-34]. Furthermore, aggressive behavior [32-34] and sexual risk taking [3-41] have been linked to aspects of impulsiveness and alcohol-induced intoxication. This might explain why only the aggression-related and sexual enhancement-related alcohol outcome expectancies were found to be associated with higher reports of engagement. Concerning readiness to change, higher ratings of impulsivity have been linked to lower stages of change [33]. Assuming that users with high levels of impulsivity engaged more often with the intervention than those with low levels, it still does not explain why they did so. On the one hand, an impulsive person might have trouble implementing the techniques taught by Vorvida and therefore might continue using the intervention to get further help. On the other hand, higher effect sizes for the number of BDDs than the average daily alcohol consumption found in the RCT [20] suggest that the intervention content might be more suitable for reducing excessive drinking than regular drinking. Therefore, impulsive users might have engaged more with the intervention due to a better fit for their needs.

### Intervention Questionnaires and Outcomes

Changes in alcohol consumption as reported in the voluntary intervention questionnaires were found to be associated with changes captured by our assessments. While larger reductions captured by the weekly questionnaire within the first 30 days were correlated only with higher reductions in average daily consumption after 180 days, higher reductions in WAC scores within the first 30 days were associated with higher reductions in daily consumption after 90 as well as 180 days. However, higher reductions captured by the daily questionnaire within the first 90 days were associated with higher reductions in average daily consumption and in the number of BDDs after 90 and 180 days.

Therefore, we could show that individual changes in drinking behavior as captured by the intervention questionnaires were associated with changes captured by our assessments. Though our analyses are only a first step toward establishing a connection between intervention questionnaires and actual behavior change, they support the idea that intervention questionnaires could potentially be used to aid dynamic tailoring. Based on data provided by the users concerning their progress, those who struggle with reducing their drinking could receive further motivational content while for those who already reduced their drinking, relapse prevention content could be provided.

### Strengths and Limitations

Our analyses are based on data that were collected as part of an RCT. Since the RCT was not designed to primarily answer questions of engagement, we have to report several limitations. SUD only captured the total time a user spent in the intervention and how often they used the questionnaires. Thus, we could not capture whether the individual’s usage was distributed over few or many sessions or which modules were completed. The fact that SUD did rarely correlate with any of the outcomes or user characteristics might therefore be due to different patterns of use resulting in the same amount of time spent in the intervention [7,12]. Because the questionnaire that we used to measure SRU has not been evaluated, its validity remains uncertain. At the time the questionnaire was developed, many of the central works that advanced the theoretical understanding of engagement had not yet been published, eg, the work of Yeager and Benight [14]. Therefore, some important concepts of engagement could not be considered in the context of operationalization of engagement. Furthermore, since participants were asked to report their intervention usage over the previous 3 months, answers might be influenced by recall bias [1], which could limit the validity of the results. This limitation of the validity of the results could be an alternate explanation for the discrepancy between subjective and objective usage. This limitation of the validity of the results could be an alternate explanation for the discrepancy between subjective and objective usage. In addition to this, as social desirability bias is a potential problem for all self-report measures [42], our results might be influenced by social desirability. Nonetheless, our study is one of the few studies to analyze relationships between engagement and changes in drinking behavior utilizing SRU as well as SUD to measure engagement. Furthermore, we provide analyses of relationships between engagement and a wide range of different demographic, clinical, and psychological variables based on a fairly large sample.

### Conclusions

Overall, our results concerning engagement-outcome relationships and associations between user characteristics and engagement add to the inconclusive evidence that can be found across the literature [1,8,14]. SRU, unlike SUD, was consistently related to better outcomes. This suggests that our questionnaire also captured other dimensions of engagement apart from the time spent using the intervention and that these dimensions are crucial for behavior change. On top of this, we found SRU to be associated with multiple, intercorrelated user characteristics, most of which have been linked to impulsivity in the past. This indicates that Vorvida might be especially engaging to impulsive persons. However, since DBCIs are made available for a wide range of target behaviors, with different content and features, it is uncertain whether this result can be generalized to other DBCIs. Finally, we could show that changes in the target behavior as captured by intervention questionnaires were related to changes reported in our assessments.

### Recommendations for Future Research

As evidence for the efficacy of DBCIs grows, it becomes more important to explore the question of how DBCIs bring about behavior change. A key part of this endeavor will be to gain a better understanding of the various facets of engagement and how they relate to behavior change. Different frameworks and theoretical models have recently been proposed to guide engagement-based research [1,6,14]. To empirically test these models, it will be crucial to measure the multidimensional nature of engagement in a reliable and valid way. Therefore, psychometrically sound self-report questionnaires that capture aspects of the micro and macro level as well as qualitative aspects of engagement need to be developed and tested in future studies. Since it might be difficult to remember usage behavior over an extended period, we encourage timely assessment of the intensity of use. This would allow measurement of engagement and early responses to the intervention in the moment, avoid recall bias, and could potentially advance tailoring. To identify universal predictors of engagement, it appears reasonable to shift the focus from demographic and problem behavior–specific variables to theory-based constructs as Yeager and Benight have suggested [14].

## Appendix

Multimedia Appendix 1 Overview of all measures used in the analyses.

Multimedia Appendix 2 Correlations between measures of engagement and changes in drinking behavior.

Multimedia Appendix 3 Correlations between clinical as well as psychological baseline characteristics and measures of engagement.

## Abbreviations

AUDIT-C: Alcohol Use Disorders Identification Test-Concise
BDD: binge drinking day
DAC: daily alcohol check
DBCI: digital behavior change intervention
FMB: five-minute blocks of activity
MC: mood check
RCT: randomized controlled trial
SRU: self-reported usage
SUD: system usage data
TFB: Timeline Followback
WAC: weekly alcohol check
