# Supplementary material for: Engagement With a Web-Based Intervention to Reduce Harmful Drinking: Secondary Analysis of a Randomized Controlled Trial
Source: J Med Internet Res. 2020 Nov 20;22(11):e18826. doi: 10.2196/18826 (PMC7718095; doi:10.2196/18826)
Supplement: Multimedia Appendix 1 [file jmir_v22i11e18826_app1.pdf]

Overview of all measures used in the analyses

Measure	Reference	Abbreviation	Construct	No. of Items	Description	Time points measured		
						**T0**	**T1**	**T2**
Timeline-Follow-Back	[23]	TFB	Drinking Behavior	7	Average daily alcohol consumption within the last 7 days. E.g. "How many glasses of which beverage did you drink last Monday?"	**X**	**X**	**X**
Binge-Drinking-Days		BDD	Drinking Behavior	1	Number of binge-drinking-days within the last 30 days. E.g. "On how many days within the past 30 days did you drink more than five drinks on one occasion?"	**X**	**X**	**X**
Five-Minute-Blocks		FMB	Engagement	/	Server-log data measuring the time spent using the intervention in blocks of five minutes. Measured continuously and summed up for the first and second 90 days of the usage period.		**X**[a]	**X**[a]
Daily Alcohol Check		DAC	Engagement / Drinking Behavior	1	Intervention questionnaire measuring the number of standard units of alcoholic drinks consumed yesterday.<br><br>**Item**: "How much did you drink yesterday?"<br>12 Options: "Nothing"– „More than 5 standard drinks"	**X**[a]	**X**[a]	**X**[a]
Weekly Alcohol Check		WAC	Engagement / Drinking Behavior	2	Intervention questionnaire measuring the number of standard units of alcoholic drinks consumed last week.<br><br>**Items**:<br>• "During the last week: On how many days did	**X**[a]	**X**[a]	**X**[a]

				you drink alcohol?”<br>8 Options: “None“ - „On 7 days“<br>• “During the last week: On a drinking day, how much did you drink on average?”<br>7 Options: “1 or 2 standard drinks“ – “More than 12 standard drinks“			
Mood Check	MC	Engagement	6	Intervention questionnaire measuring the current mood on five-point Likert scales. E.g. “Today, so far I feel stressed and frustrated.”		**X**[a]	**X**[a]
Self-reported usage	SRU	Engagement	1	Intervention usage assessed by self-report.<br>**Item:** “Please state how often you used Vorvida within the last three months?”<br>Options: “Weekly more than 4 hours”, “Weekly 3 – 4 hours”, “Weekly 2 – 3 hours”, “Weekly less than 2 hours”, “Weekly less than 1 hour”, “I didn’t use Vorvida at all”.		**X**	**X**
Sociodemographic Data		User characteristic	7	Different socio-demographic data on the participant, including **age**, **gender** (female / male), **relationship status** (single / non-single), **children** (one ore more / no children), **education** (General higher education entrance qualification (Abitur) / no general higher education entrance qualification (No Abitur)), **job qualification** (academic studies / no academic studies) and **job status** (working / not working)	**X**		

Age of first alcohol consumption		AFC	User characteristic	1	Age of the participant, when they first consumed alcohol.	**X**
Age of regular alcohol consumption		ARC	User characteristic	1	Age of the participant, when they started to consume alcohol on a regular basis.	**X**
Baseline Drinking Frequency	[23]	QFI-Days	User characteristic	1	Days of alcohol consumption within the last 30 days, measured as part of the Quantity-Frequency-Index.<br><br>**Item**: "On how many days within the last 30 days did you drink alcohol?"	**X**
Readiness Ruler	[25]	RR	User characteristic	2	Participants ratings on 10-point scales of how important it is to them to drink less (**RR-I**) and of how confident they are to achieve a reduction (**RR-C**).	**X**
Alcohol abstinence self-efficacy scale	[26]	AASE	User characteristic	40	Participants rating on two scales with the same 20 items, describing different situations, e.g. "When I am feeling angry inside.".<br><br>**Two Subscales**: Temptation (**AASE-T**) to drink in given situations, rated on a 5-Point Likert scale ("No temptation at all" - "Very strong temptation") and Confidence (**AASE-C**) not to drink in given situation, rated on a 5-Point Likert scale ("Not confident at all" - "Absolutely confident")	**X**
Comprehensive Alcohol Expectancy Questionnaire	[27]	CAEQ	User characteristic	19	Self-assessment of the expectation of the effects of alcohol on five different subscales, where every Item is prefaced with "When I drink alcohol…". E.g. "I am more	**X**

					relaxed and more at ease socially”<br><br>**Five Subscales**: 5-point Likert scales (“not at all” - “definitely”) measuring expectations concerning the topics of aggression (**CAEQ-A**), cognitive impairment and physical discomfort (**CAEQ-CP**), sexual enhancement (**CAEQ-SE**), social assertiveness and positive affect (**CAEQ-SP**) and tension reduction (**CAEQ-T**).	
Readiness to Change Questionnaire	[28]	RCQ	User characteristic	12	Participants rating of their readiness to change on three subscales, representing the three original stages of change. E.g. “I enjoy my drinking, but sometimes I drink too much”<br><br>**Three Subscales**: 5-point Likert scales (“don’t agree at all” - “agree a lot”) with items representing the Precontemplation stage (**RCQ-P**), the contemplation stage (**RCQ-C**) and the Action stage (**RCQ-A**)	**X**

[a] Data was measured continuously, but aggregated to match time points.
