# Supplementary material for: Engagement With a Web-Based Intervention to Reduce Harmful Drinking: Secondary Analysis of a Randomized Controlled Trial
Source: J Med Internet Res. 2020 Nov 20;22(11):e18826. doi: 10.2196/18826 (PMC7718095; doi:10.2196/18826)
Supplement: Multimedia Appendix 2 [file jmir_v22i11e18826_app2.pdf]

Correlations between measures of engagement and changes in drinking behavior

(r/ρ) \ n	1.	2.	3.	4.	5.	6.	7.	8.	9.	10.	11.	12.	13. [a]	14. [a]	15.	16.	17.	18.	19.	20.
**1. FMB T1**	-	306	306	306	306	306	306	306	306	306	306	306	200	183	204	187	189	306	306	306
**2. FMB T2**	.5***	-	306	306	306	306	306	306	306	306	306	306	200	183	204	187	189	306	306	306
**3. FMB TOT**	.97***	.71***	-	306	306	306	306	306	306	306	306	306	200	183	204	187	189	306	306	306
**4. DAC T1**	.93***	.62***	.94***	-	306	306	306	306	306	306	306	306	200	183	204	187	189	306	306	306
**5. DAC T2**	.47***	.98***	.68***	.59***	-	306	306	306	306	306	306	306	200	183	204	187	189	306	306	306
**6. DAC TOT**	.83***	.85***	.93***	.94***	.84***	-	306	306	306	306	306	306	200	183	204	187	189	306	306	306
**7. WAC T1**	.82***	.35***	.77***	.77***	.29***	.65***	-	306	306	306	306	306	200	183	204	187	189	306	306	306
**8. WAC T2**	.47***	.86***	.64***	.56***	.86***	.75***	.39***	-	306	306	306	306	200	183	204	187	189	306	306	306
**9. WAC TOT**	.83***	.57***	.84***	.82***	.52***	.78***	.95***	.65***	-	306	306	306	200	183	204	187	189	306	306	306
**10. MC T1**	.65***	.47***	.67***	.73***	.47***	.7***	.27***	.48***	.38***	-	306	306	200	183	204	187	189	306	306	306
**11. MC T2**	.41***	.74***	.56***	.52***	.73***	.67***	.17	.69***	.37***	.67***	-	306	200	183	204	187	189	306	306	306
**12. MC TOT**	.62***	.59***	.68***	.72***	.59***	.75***	.26***	.58***	.41***	.97***	.83***	-	200	183	204	187	189	306	306	306
**13. SRU T1 [a]**	.29**	.14	.3**	.29**	.19	.24	.44***	.11	.25	-.26*	-.34***	-.27*	-	179	199	184	184	200	200	200
**14. SRU T2 [a]**	-.04	.24	-.03	.04	.2	.08	.38***	.01	.15	-.34***	-.4***	-.34***	.5***	-	181	181	183	183	183	183
**15. BDD T1**	.14	-.19	.05	-.01	-.17	-.08	.26*	-.23	.13	-.24	-.18	-.24	.62***	.27*	-	187	187	204	204	204
**16. BDD T2**	-.07	-.03	-.07	-.02	-.05	-.03	.17	-.05	.12	-.17	-.06	-.14	.05	.3**	-.38***	-	187	187	187	187
**17. BDD TOT**	.04	-.24	-.05	-.07	-.22	-.15	.36***	-.28*	.18	-.39***	-.24	-.37***	.65***	.42***	.68***	.42***	-	189	189	189
**18. TFB T1**	.02	-.1	-.02	-.03	-.1	-.07	.01	-.06	-.01	-.06	-.04	-.05	.39***	.09	.42***	-.09	.37***	-	306	306
**19. TFB T2**	-.17	-.06	-.16	-.13	-.06	-.12	-.1	-.08	-.1	-.15	-.07	-.14	.17	.29*	.01	.31**	.32**	.33***	-	306
**20. TFB TOT**	-.09	-.1	-.1	-.1	-.1	-.11	-.05	-.08	-.07	-.12	-.07	-.11	.42***	.22	.33***	.13	.58***	.84***	.79***	-

Note. Correlation coefficients are displayed below the diagonal; Number of cases are displayed above the diagonal; [a] Spearman correlation coefficients are displayed; FMB, Usage of the intervention measured as five minute blocks; DAC, Frequency of utilization of the Daily Alcohol Check; WAC, Frequency of the utilization of the Weekly Alcohol Check; MC, Frequency of the utilization of the Mood Check; SRU, Self-reported usage of the Intervention; BDD, Reductions in binge drinking days; TFB, Reductions in daily alcohol consumption (Timeline Follow-Back); T1, Period from baseline to first post-assesment (90 days); T2, Period from first to second post-assesment (90 days); TOT, Total usage period (180 days); *, $P \le .05$; **, $P \le .01$; ***, $P < .001$
