# Supplementary material for: Engagement With a Web-Based Intervention to Reduce Harmful Drinking: Secondary Analysis of a Randomized Controlled Trial
Source: J Med Internet Res. 2020 Nov 20;22(11):e18826. doi: 10.2196/18826 (PMC7718095; doi:10.2196/18826)
Supplement: Multimedia Appendix 3 [file jmir_v22i11e18826_app3.pdf]

Correlations between clinical as well as psychological baseline characteristics and measures of engagement

(r/ρ) \ n	1.	2.	3.	4.	5.	6. [a]	7. [a]	8.	9.	10.	11.	12.	13.	14.	15.	16.	17.	18. [a]	19. [a]	20.	21.
**1. AFC**	-	306	306	304	306	296	296	298	296	299	299	299	299	299	298	298	298	200	183	306	306
**2. ARC**	.62***	-	306	304	306	296	296	298	296	299	299	299	299	299	298	298	298	200	183	306	306
**3. QFI-Days**	.22*	.05	-	304	306	296	296	298	296	299	299	299	299	299	298	298	298	200	183	306	306
**4. BDD**	.06	-.18	.68***	-	304	296	296	298	296	299	299	299	299	299	298	298	298	200	183	304	304
**5. TFB**	-.13	-.14	.14	.15	-	296	296	298	296	299	299	299	299	299	298	298	298	200	183	306	306
**6. RR-I [a]**	.03	.02	.10	.04	-.01	-	296	296	296	296	296	296	296	296	296	296	296	199	181	296	296
**7. RR-C [a]**	-.03	-.06	-.07	.14	.05	-.05	-	296	296	296	296	296	296	296	296	296	296	199	181	296	296
**8. AASE-T**	.04	-.16	.4***	.41***	.15	.22*	-.21	-	298	298	298	298	298	298	298	298	298	199	182	298	298
**9. AASE-C**	-.12	.07	-.29***	-.24*	.04	-.19	.36***	-.6***	-	296	296	296	296	296	296	296	296	199	181	296	296
**10. CAEQ-A**	.08	-.01	.23*	.44***	.04	.15	.12	.4***	-.16	-	299	299	299	299	298	298	298	199	182	299	299
**11. CAEQ-CP**	-.02	-.11	.01	.21	.00	.17	-.12	.49***	-.32***	.47***	-	299	299	299	298	298	298	199	182	299	299
**12. CAEQ-SE**	.17	-.10	.25**	.38***	.00	-.01	.08	.43***	-.18	.5***	.33***	-	299	299	298	298	298	199	182	299	299
**13. CAEQ-SP**	.08	-.21	-.08	.06	-.09	.05	-.09	.4***	-.27***	.20	.36***	.5***	-	299	298	298	298	199	182	299	299
**14. CAEQ-T**	.00	-.03	-.05	-.02	.06	.27***	-.16	.43***	-.26**	.23*	.47***	.24**	.39***	-	298	298	298	199	182	299	299
**15. RCQ-C**	-.22*	.02	-.14	-.24**	.11	.48***	-.23*	.13	-.03	-.03	.16	-.20	.05	.38***	-	298	298	199	182	299	299
**16. RCQ-P**	.21	-.05	.16	.31***	-.01	-.51***	.21	.05	.02	.18	-.01	.37***	.12	-.29***	-.78***	.-	298	199	182	299	299

**17. RCQ-A**	-.08	-.11	.01	.13	-.02	.4***	.2	.3***	-.03	.36***	.25**	.21	.12	.26**	.29***	-.22*	-	199	182	299	299
**18. SRU T1** [a]	.34***	-.05	.37***	.52***	.26	-.16	.26*	.19	-.05	.34***	.17	.38***	-.02	-.19	-.43***	.57***	-.07	-	179	200	200
**19. SRU T2** [a]	.43***	-.01	.6***	.56***	.11	-.03	.04	.46***	-.15	.47***	.25	.54***	.08	-.04	-.31**	.43***	.09	.05***	-	183	183
**20. FMB T1**	.09	-.01	.29***	.27***	-.09	.06	.19	.12	-.09	.08	.08	-.04	.02	-.01	.04	-.02	.14	.29**	-.04	-	306
**21. FMB T2**	.01	.06	.05	-.04	-.08	.07	-.07	.11	-.09	-.03	.05	-.13	-.07	.03	.12	-.11	.02	.14	.24	.5***	-

Note. Correlation coefficients are displayed below the diagonal; Number of cases are displayed above the diagonal; [a] Spearman correlation coefficients are displayed; AFC, Age of first alcohol consumption; ARC, Age of regular alcohol consumption; QFI-Days: Drinking days measured with the Quantitiy-Frequency-Index; BDD, Binge Drinking Days; TFB, Average daily alcohol consumption measured with the Timeline-Followback approach; RR, Readiness Ruler; RR-I, Importance scale of RR; RR-C, Confidence scale of RR; AASE, Alcohol Abstinence Self-Efficacy Scale; AASE-T, Temptation Scale of the AASE; AASE-C, Confidence Scale of the AASE; CAEQ, Comprehensive Alcohol Expectancy Questionnaire; CAEQ-A, Aggression scale of the CAEQ; CAEQ-CP, Cognitive impairment and physical discomfort scale of the CAEQ; CAEQ-SE, Sexual enhancement scale of the CAEQ; CAEQ-SP, Social assertiveness and positive affect scale of the CAEQ; CAEQ-T, Tension reduction scale of the CAEQ; RCQ, Readiness to Change Questionnaire; RCQ-C, Contemplation scale of the RCQ; RCQ-P, Precontemplation scale of the RCQ; RCQ-A, Action scale of the RCQ; SRU, Self-reported usage; FMB, Usage of the Intervention measured as five minute blocks; T1, Period from baseline to first post-assesment (90 days); T2, Period from first to second post-assesment (90 days); *, $P \le .05$; **, $P \le .01$; ***, $P < .001$
